# Transcutaneous interference spinal cord stimulation: leadfield-based pareto optimization of electrode montages for improved focality

**DOI:** 10.1007/s13534-025-00531-2

**Published:** 2025-11-17

**Authors:** Mariko Teragiwa, Leonel E. Medina, Alonso Carvajal, Kanata Yatsuda, Wenwei Yu, Jose Gomez-Tames

**Affiliations:** 1 Department of Medical Engineering, Graduate School of Engineering, Chiba University, Chiba, 263-8522 Japan; 2 Facultad de Ingeniería, Universidad de Santiago de Chile, Avda. Víctor Jara, 3659 Santiago, Chile; 3 Center for Frontier Medical Engineering, Chiba University, Chiba, 263-8522 Japan

**Keywords:** Transcutaneous spinal cord stimulation, Temporal interference, Electrode montage, Leadfield, Pareto optimization

## Abstract

This study investigates the feasibility of transcutaneous interferential spinal cord stimulation (tISCS), a novel non-invasive neuromodulation method, using temporal interference to enhance focality and comfort in spinal cord stimulation. The central research question is whether tISCS can achieve targeted activation of spinal cord circuits while reducing unwanted stimulation of skin and muscle tissues, which are common limitations of conventional transcutaneous spinal cord stimulation (tSCS). A finite element model of the lower thorax was developed to simulate electric field distributions for various skin electrode montages. To address the computational bottleneck associated with high-resolution modeling and montage optimization, we implemented a leadfield-based Pareto optimization strategy to identify the electrode configuration that maximizes the electric field in the spinal cord and minimizes it in off-target tissues. tISCS montages were compared with tSCS montages in terms of focality and stimulation efficiency. Optimized tISCS configurations significantly reduced electric field intensity in the skin by over 20-fold compared to tSCS. The ratio of spinal cord to skin electric fields increased by at least tenfold, indicating enhanced focality. The injection current efficiency in tISCS can be leveraged to increase spinal cord electric fields by at least fivefold while keeping skin exposure below the levels observed with tSCS. tISCS enables deeper and more selective spinal cord stimulation compared to tSCS, with substantially reduced off-target effects. In conclusion, this is the first computational demonstration of tISCS feasibility, and leadfield-guided Pareto optimization enables efficient montage selection, providing a foundation for future experimental applications.

**Supplementary Information:**

The online version contains supplementary material available at 10.1007/s13534-025-00531-2.

## Introduction

One method for spinal cord stimulation involves implanting electrodes in the epidural space that deliver controlled electrical pulses to targeted areas of the spinal cord, known as epidural spinal cord stimulation (eSCS). eSCS is used to treat chronic and neuropathic pain and is being investigated to restore mobility for individuals with cervical spinal cord injuries [1, 2]. Despite its significant benefits, one challenge with eSCS is the inherent risk associated with implanting the stimulation leads and the pulse generator [3, 4]. Moreover, the placement of the stimulation leads may shift over time, potentially leading to unintended stimulation and diminishing the therapy's effectiveness. Another concern is that the leads could break or bend, resulting in a malfunction [5].

An alternative strategy is transcutaneous spinal cord stimulation (tSCS), which delivers electric current through surface electrodes placed on the skin over the spine, typically in the thoracolumbar region, generating an electric field intended to modulate spinal circuits [6]. However, tSCS is limited in its ability to precisely target specific neural structures due to substantial current attenuation at depth, resulting in low focality. Achieving sufficient intensity at the spinal cord requires high surface currents, which can activate cutaneous nociceptors-thereby causing pain-and unintentionally stimulate nearby organs, including muscles [6]. Therefore, minimizing activation or modulation of cutaneous nociceptors, sensory fibers, and/or motor fibers while maintaining effective spinal stimulation is a critical challenge.

One transcutaneous stimulation modality is the so-called temporal interference stimulation [7, 8]. Temporal interference applies two slightly different kilohertz (kHz) currents via distinct electrode pairs *(e.g., 2 kHz and 2.01 kHz)*. Their linear superposition produces amplitude-modulated electric field whose low-frequency envelope equals the frequency difference between the two kHz waveforms. The modulation depth of the low-frequency envelope is the difference between the maximum and minimum values of the envelope and describes the neuromodulation level of temporal interference [8, 9]. It is argued that neuronal membranes exhibit low-pass filtering properties together with nonlinear membrane rectification mediated by voltage-gated ion channels [8, 10]. Thus, modulation of nerve fiber activity is occurring at the frequency of the envelope of the interference signal (low frequency component). By properly selecting the electrode positions, it is possible to produce a low-frequency envelope with high modulation depth in the deep target and a low modulation depth outside. Therefore, superficial areas are apparently predominantly influenced by the high-frequency components of the stimulation signals, which would result in no activation effects and/or conduction block on cutaneous afferents [10]. In the brain, temporal interference has been shown to modulate deep brain regions and is well tolerated [11]. For peripheral nerves, temporal interference stimulation allows higher injected currents without exceeding pain thresholds, enabling deeper stimulation with improved comfort [12, 13].

However, it remains unclear whether temporal interference stimulation can effectively target the spinal cord with sufficient focality while minimizing cutaneous pain and muscle activation. This uncertainty presents a significant obstacle to translating temporal interference into SCS applications. A promising strategy for addressing whether temporal interference stimulation can effectively target the spinal cord is the utilization of computational modeling of electric fields in anatomically based models. Such models have proven valuable for optimizing montages in tSCS [14–16], as well as for investigating temporal interference in the brain [17]. Yet, to date, no studies have applied computational modelling specifically to transcutaneous interference spinal cord stimulation (tISCS). A major limitation in applying these models to tISCS is the substantial computational cost associated with high-resolution spinal cord models (e.g., 2.5 hours per montage [18]), which increases dramatically when optimizing. To overcome this bottleneck, the leadfield matrix approach offers an effective solution in neurostimulation [19, 20]. This approach involves precomputing and storing the electric field distributions generated by a set of electrodes pairs. Once this matrix is established, the resulting electric field for any arbitrary combination of the electrodes in the set can be rapidly obtained via linear combinations of the precomputed fields, enabling fast and efficient simulation of numerous stimulation configurations in temporal interference for the brain [21, 22]. Extending these modelling frameworks to tISCS would offer a critical foundation for exploring field distributions, targeting specificity, and enabling informed design of stimulation protocols before clinical implementation.

This study aims to computationally explore the feasibility of tISCS, focusing on its potential to reduce unwanted effects on the skin and muscle and to provide a more effective targeted approach. To this end, we estimated the electric field using an anatomically based finite element model of the thorax and optimized the electrode montage based on the Pareto front.

## Methodology

### Human model design

**Fig. 1:**
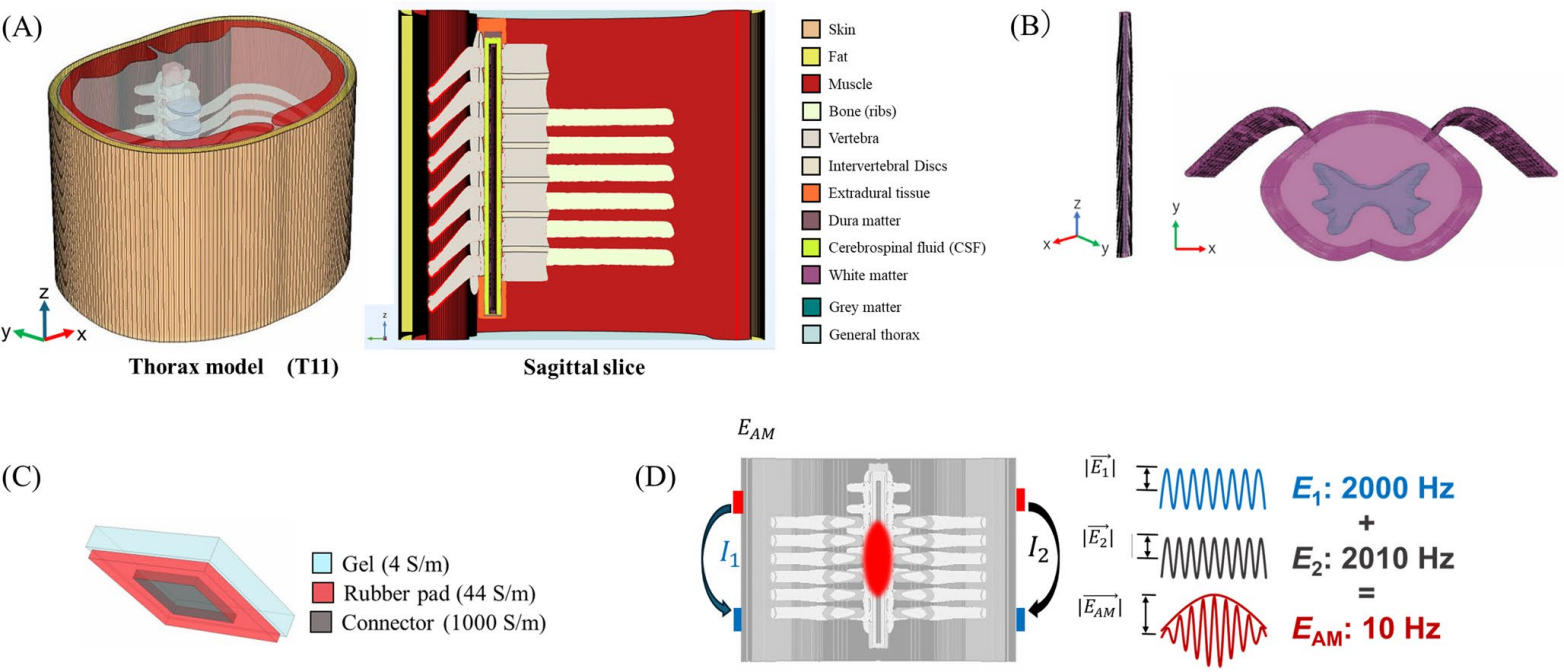
Human and electrode model. **A** The whole thorax model and each tissue, **B** the model of the spinal cord, **C** the model of electrode, **D** transcutaneous interference spinal cord stimulation, or tISCS

A realistic finite element method (FEM) model of the thorax was constructed based on anatomical data from the lower thoracic spinal level (T11) [23] and surrounding anatomical tissues [24, 25]. The model was segmented into 12 tissues, including skin, fat, muscle, general thorax, bone, vertebrae, intervertebral disks, extradural tissue, dura matter, cerebrospinal fluid (CSF), spinal white matter (spinal-WM), and spinal gray matter (spinal-GM), as shown in the Fig. 1A and B The anatomical structures were represented as STL files and imported into COMSOL using its native geometry import functions. The mesh was then manually revised to eliminate intersections and ensure continuous interfaces between tissues.

The electrical conductivity values were set according to Table 1 [26–30]. A conductivity tensor was implemented to account for the anisotropic nature of spinal-WM, where conductivity is higher along the caudal-to-rostral axis [31]. tISCS operates at carrier frequencies of a few kilohertz, whereas tSCS typically uses frequencies below 100 Hz. While tissue conductivity is known to be frequency-dependent, we adopted the conductivity values commonly reported for tSCS. This approximation is justified, as prior study has shown that variations in conductivity within this frequency range have a negligible impact on tISCS [32].

**Table 1:** Tissue electrical conductivity in the human thorax model

Tissue	Conductivity (S/m)	Reference
Skin	0.148	[26]
Fat	0.077	[26]
Muscle	0.355	[48]
General thorax (water)	0.250	[49]
Bone / Vertebrae	0.006	[26]
Intervertebral disks	0.650	[28]
Extradural tissue	0.250	[28]
Dura matter	0.461	[29]
CSF	1.880	[26]
Spinal-WM (transverse)	0.083	[30]
Spinal-WM (longitudinal)	0.600	[30]
Spinal-GM	0.230	[30]

### Electrodes model

A two-layered electrode was used. The layer on top is silicone rubber (2401 mm^2^, 44 S/m [33]). The bottom layer consists of a saline gel (2500 mm^2^, 4 S/m [33]). It also includes a rectangular connector (108 mm^2^, 1000 S/m) affixed underneath the rubber pad (Fig. 1C).

### Electric field model

Using the quasi-static approximation and the finite element technique (COMSOL Multiphysics Ver. 6.2) to solve Laplace’s equation [34], the electric potential produced by the current injected from a pair of electrodes (common bipolar tSCS) placed on the thorax was estimated using the following equation:

1 $$\begin{array}{*{20}c} {\nabla \cdot \left( {\sigma \nabla \varphi } \right) = 0,} \\ \end{array}$$

where *φ* and *σ* denote the scalar potential and tissue conductivity, respectively. The electric field $\overrightarrow{E}$=-∇φ was calculated in each element. There were 7,139,950 tetrahedral elements. The top 0.01% outliers were removed from the sorted E-field intensity produced by potential mesh errors [35]. The results were numerically stable relative to a finer mesh.

For bipolar tSCS, the electric field strength was obtained for a pair of electrodes. In the case of tISCS (Fig. 1D), the amplitude-modulated envelope (*E*_AM_) is generated from the vector sum of the two resultant electric fields ($\overrightarrow{{E}_{1}}$ and $\overrightarrow{{E}_{2}}$), each corresponding to a pair of electrodes driven by an independent current source. The electric fields at location $\overrightarrow{r}$ are represented by $\overrightarrow{{E}_{1}}$ ($\overrightarrow{r}$) and $\overrightarrow{{E}_{2}}$ ($\overrightarrow{r}$). If |$\overrightarrow{{E}_{1}}$| >|$\overrightarrow{{E}_{2}}$| and the angle $\alpha$ (angle between |$\overrightarrow{{E}_{1}}$| and |$\overrightarrow{{E}_{2}}$|) is lower than 90°, the modulation depth is expressed by [8, 17]

2 $$\begin{gathered} \left| {\vec{E}_{{AM}} \left( {\vec{r}} \right)} \right| \hfill \\ \quad = \left\{ {\begin{array}{*{20}c} {2\left| {\vec{E}_{2} \left( {\vec{r}} \right)} \right|} & {if\left| {\vec{E}_{2} \left( {\vec{r}} \right)} \right| < \left| {\vec{E}_{1} \left( {\vec{r}} \right)} \right|cos\alpha } \\ {2\left| {\vec{E}_{2} \left( {\vec{r}} \right) \times \frac{{\left( {\vec{E}_{1} \left( {\vec{r}} \right) - \vec{E}_{2} \left( {\vec{r}} \right)} \right)}}{{\left| {\vec{E}_{1} \left( {\vec{r}} \right) - \vec{E}_{2} \left( {\vec{r}} \right)} \right|}}} \right|} & {otherwise} \\ \end{array} } \right. \hfill \\ \end{gathered}$$

The direction of one vector must be flipped if the angle is greater than 90°. The Eq. (3) provides an expression that uses the EF vector (${E}_{x}\left(\overrightarrow{r}\right)$, ${E}_{y}\left(\overrightarrow{r}\right)$, ${E}_{z}\left(\overrightarrow{r}\right)$) to obtain the maximum intermodulation depth intensity, corresponding to the direction of maximum modulation [36].

For the conventional tSCS, we used a bipolar montage with a midline orientation and inter-electrode distance of 2.5 cm [33, 37]. The electrode positions for tISCS were determined through a quasi-optimization process, described in the next subsection. The total injected current was fixed at 2.5 mA for conventional tSCS and tISCS [38]. As a result, the current per electrode pair in tISCS is halved relative to conventional tSCS. To ensure a fair comparison, we also implemented a two-pair in-phase bipolar tSCS configuration (referred to as 2-tSCS), matching both the number of electrodes and the current density used in tISCS. The electrode positions for 2-tSCS were set identically to those used for tISCS. It is important to note that the choice of maximum injected current does not affect the generalizability of the results, as the electric field magnitude in the model can be scaled linearly with the injection current intensity. Finally, we included an intuition-based montage for tISCS that was not derived from the optimization process but was instead adapted from conventional tSCS, using the same inter-electrode distance [33]. This montage serves as a baseline reference that might reasonably be chosen in practice without computational guidance.

### Leadfield matrix

The leadfield matrix ${{\boldsymbol{A}}}_{{\boldsymbol{n}}}$ is a linear mapping from the injected current ${{\boldsymbol{x}}}_{{\boldsymbol{n}}}$ to the resulting electric field $\overrightarrow{E} \left(p\right)$ at mesh node $p$:

3 $$\overrightarrow{E} \left(p\right)={{\boldsymbol{A}}}_{{\boldsymbol{n}}}{{\boldsymbol{x}}}_{{\boldsymbol{n}}}.$$

To build the precomputed leadfield matrix ${{\boldsymbol{A}}}_{{\boldsymbol{n}}}$, an electrode was fixed as the return in the center of the thorax, and a unit current was sequentially injected through each electrode distributed around the thorax for a total of 162 electrodes (6 vertical $\times$ 27 horizontal positions). The simulations were carried out in COMSOL Multiphysics (v6.2) on an Intel(R) Xeon(R) W-2295 processor (3.00 GHz, 128 GB RAM), requiring roughly 30 h of computation. The resulting matrix, with dimensions corresponding to 162 montages × 3 field components × 7,139,750 mesh elements, enables rapid prediction of the electric field $\overrightarrow{E} \left(p\right)$ for any linear combination of input currents ${{\boldsymbol{x}}}_{{\boldsymbol{n}}}$.

### Optimization method

**Fig. 2:**
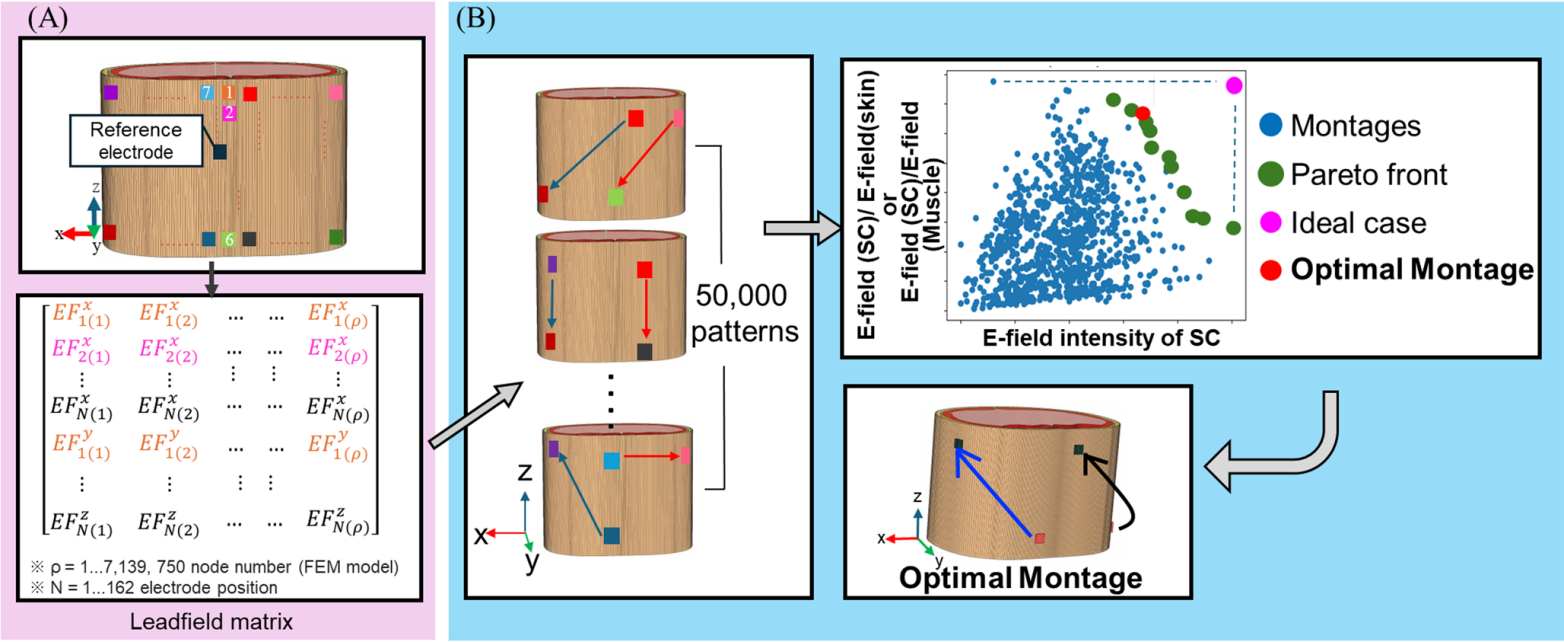
Optimisation procedure. **A** A total of 162 electrodes are used to build the leadfield matrix, **B** amplitude-modulated envelope (*E*_AM_) are obtained from a total of 50,000 random electrode combinations using the leadfield matrix. A Pareto front is formed from the trade-off between the maximum intensity of *E*_AM_ in the spinal cord and the ratio of *E*_AM_ between the spinal cord and skin (or muscle)

In the first step, we performed a brute-force search over a randomly sampled subset of montages candidates (quasi-optimization) to enable unbiased global exploration, ensuring that potentially effective electrode placements around the thorax were not overlooked. Specifically, 50,000 montages were sampled from a grid of 162 candidate electrode positions surrounding the thorax (Sect. 3.1). Two metrics guided the optimization: maximizing target intensity and maximizing focality [17, 39]. These objectives showed a trade-off that formed a Pareto front, consistent with prior findings (Fig. 2B). The Supplementary Material shows that quasi-optimization is stable for subspaces of more than 10,000 montages.

In the second step, we constructed a reduced subspace by retaining the most influential electrodes identified during global exploration (the “relevant electrode map,” Sect. 2.6), and then exhaustively evaluated all montage combinations of a 44-grid positions, as shown in Sect. 3.2.

From Eq. (3), the target intensity metric was

$${\mathrm{E}}_{\mathrm{AM}(\mathrm{SC})}=\mathrm{max}\left|{\overrightarrow{\mathrm{E}}}_{\mathrm{AM}}\left(\mathrm{SC}\right)\right|,$$

using only the z-component in the spinal cord because the target dorsal column fibers are assumed to align with the z-axis [40].

Focality was defined as the ratio of spinal-cord modulation to peak modulation in off-target tissues (skin and muscle):

$${\mathrm{E}}_{\mathrm{AM}(\mathrm{SC}/\mathrm{Skin})}=\mathrm{max}\left|{\overrightarrow{\mathrm{E}}}_{\mathrm{AM}}\left(\mathrm{SC}\right)\right|/\mathrm{max}\left|{\overrightarrow{\mathrm{E}}}_{\mathrm{AM}}\left(\mathrm{Skin}\right)\right|,$$

$${\mathrm{E}}_{\mathrm{AM}(\mathrm{SC}/\mathrm{Muscle})}=\mathrm{max}\left|{\overrightarrow{\mathrm{E}}}_{\mathrm{AM}}\left(\mathrm{SC}\right)\right|/\mathrm{max}\left|{\overrightarrow{\mathrm{E}}}_{\mathrm{AM}}\left(\mathrm{Muscle}\right)\right|.$$

The terms ${\overrightarrow{E}}_{AM}\left(\mathrm{Skin}\right)$ and ${\overrightarrow{E}}_{AM}\left(\mathrm{Muscle}\right)$ represent the modulation depth along the direction of maximum modulation [36]. Because cutaneous nociceptors and muscle fibers are likely to align with this direction, these definitions yield a conservative estimate of focality.

### Relevant electrode map

**Fig. 3:**
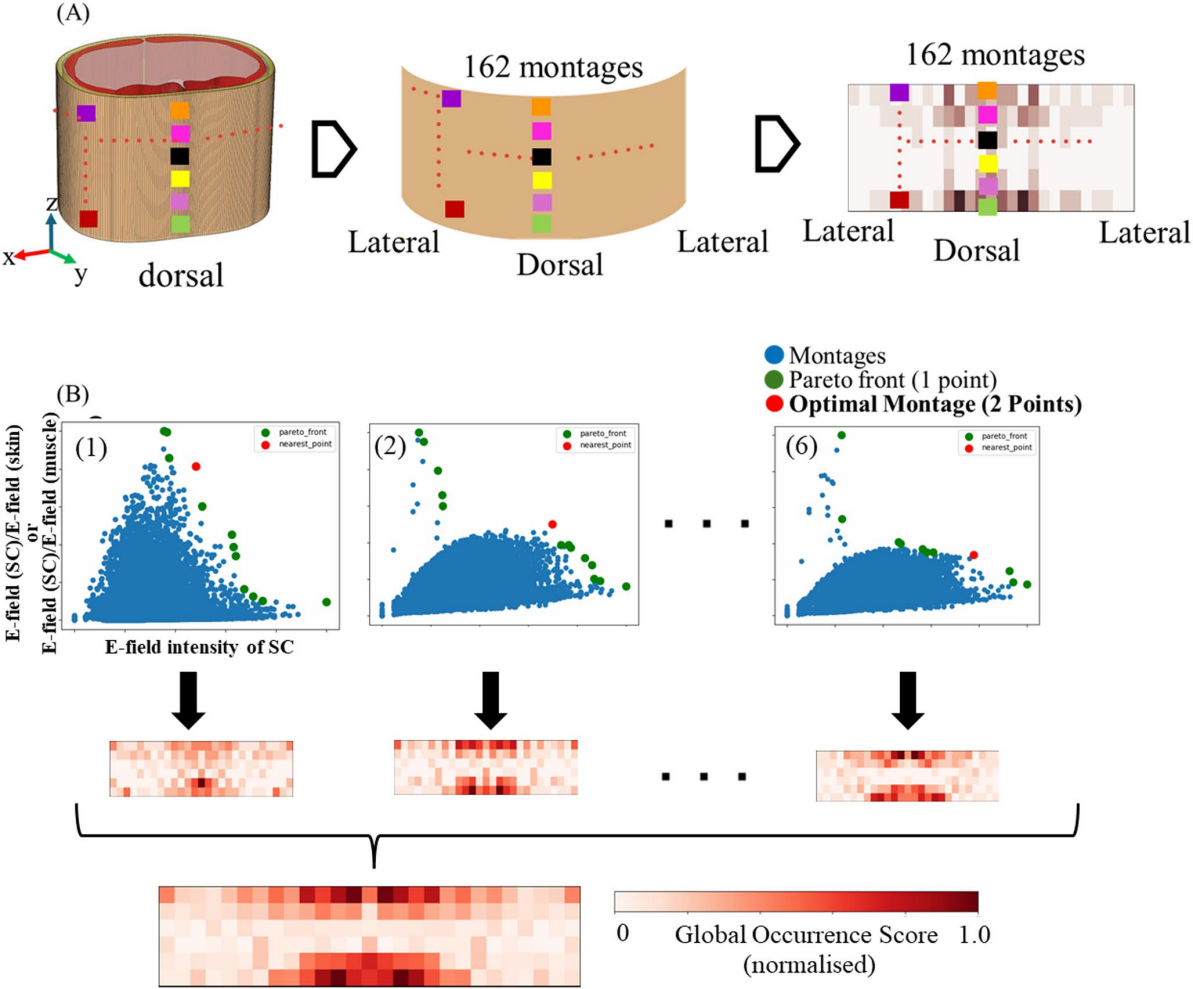
Influential electrode positions in the grid. **A** Representation of electrode positions in a plane, **B** outline to obtain the Relevant Electrode Map

We propose reducing the total number of potential electrode positions by introducing the “Relevant Electrode Map”, which shows the most influential positions. Figure 3A presents a 2D map displaying all electrode positions. Figure 3B shows the process used to identify the most relevant electrode positions. Each montage was assigned a score. A score of one point corresponds to montages on the Pareto front (green dot) derived from 50,000 random montages based on the 162-grid. A score of two points was assigned to the best-performing montage among them (red dot). These scores are accumulated over six repetitions and then normalized to generate the final Relevant Electrode Map.

## Results

### Optimization evaluation

**Fig. 4:**
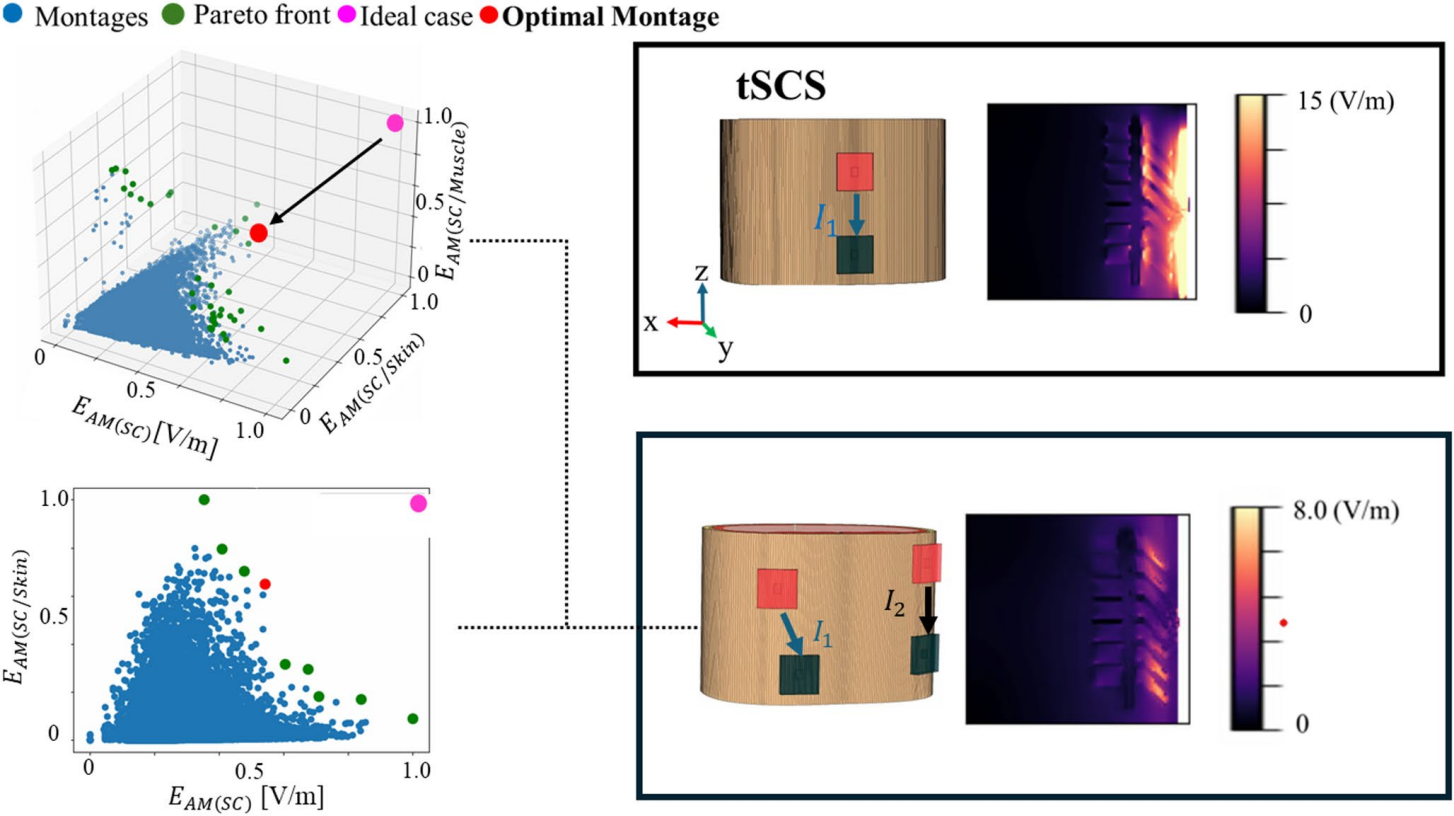
tISCS optimal montage (162-grid). Local best montage from a total of 50,000 random selected montages based on Pareto fronts (2D and 3D). As reference a common employed tSCS is shown

Figure 4 presents the optimized tISCS montage using 3D or 2D Pareto approaches using a broad search around the thorax. The 2D pareto fronts are obtained by evaluating trade-offs between the target spinal cord intensity ${\overrightarrow{E}}_{AM\left(SC\right)}$ and one of the superficial focality metrics, either ${\overrightarrow{E}}_{AM\left(SC/Skin\right)}$ or ${\overrightarrow{E}}_{AM\left(SC/Muscle\right)}$. The 3D Pareto simultaneously considered all three metrics. Results indicate that superficial electric fields are minimized more effectively using either the 3D Pareto approach or the 2D approach involving skin.

Table 2 compares optimized tISCS (3D) with 2-tSCS, which share the same electrode configuration as tISCS. The tISCS approach significantly reduces superficial ${E}_{AM}$, with reductions of 21.5-fold in the skin and 3.2-fold in the muscle compared to 2-tSCS. While the spinal cord intensity under tISCS decreases by a factor of 1.2 times, the ratios spinal cord to skin and spinal cord to muscle increase by 17.5 and 2.7 times, respectively, compared to 2-tSCS. Table 2 also includes a comparison between the optimized tISCS and conventional tSCS where the total current is the same (i.e., tISCS halves the injection current per electrode compared to tSCS). tISCS reduces peak ${E}_{AM}$ in the skin and muscle by factors of 24.7 and 6.7, respectively. Similarly, although peak ${E}_{AM}$ spinal cord decreases by a factor of 2 under tISCS, the spinal cord-to-skin and spinal cord-to-muscle ratios remain higher by 11.7 and 3.3 times, respectively, compared to tSCS. Additionally, Table 2 illustrates the difference with an ad-hoc montage (unoptimized). The optimised montage produced 3.9 times and 1.7 times better focality for ${\overrightarrow{E}}_{AM\left(SC/Skin\right)}$ and ${\overrightarrow{E}}_{AM\left(SC/Muscle\right)}$, respectively.

**Table 2:** Comparison between stimulation methods and montage optimization

Metrics	tSCS	2-tSCS	tISCS (unoptimized)	tISCS (2D) SC/skin	tISCS (2D) SC/muscle	tISCS (3D)
*Electric field [V/m]*						
Spinal cord	4.24	2.54	1.71	2.13	2.60	2.13
Skin	148.30	129.40	18.83	6.01	70.81	6.01
Muscle	22.07	10.88	4.52	3.39	4.38	3.39
*Ratio*						
Spinal cord / Skin	0.03	0.02	0.09	0.35	0.04	0.35
Spinal cord / Muscle	0.19	0.23	0.38	0.63	0.59	0.63

To assess the robustness of quasi optimization method, six independent global searches of different number of randomly selected montages showed relative standard deviations plateauing once 10,000 or more montages were sampled (see Supplementary Material), with respect to maximum electric field in the spinal cord, skin focality, and muscle focality.

### Optimization on relevant electrode positions

**Fig. 5:**
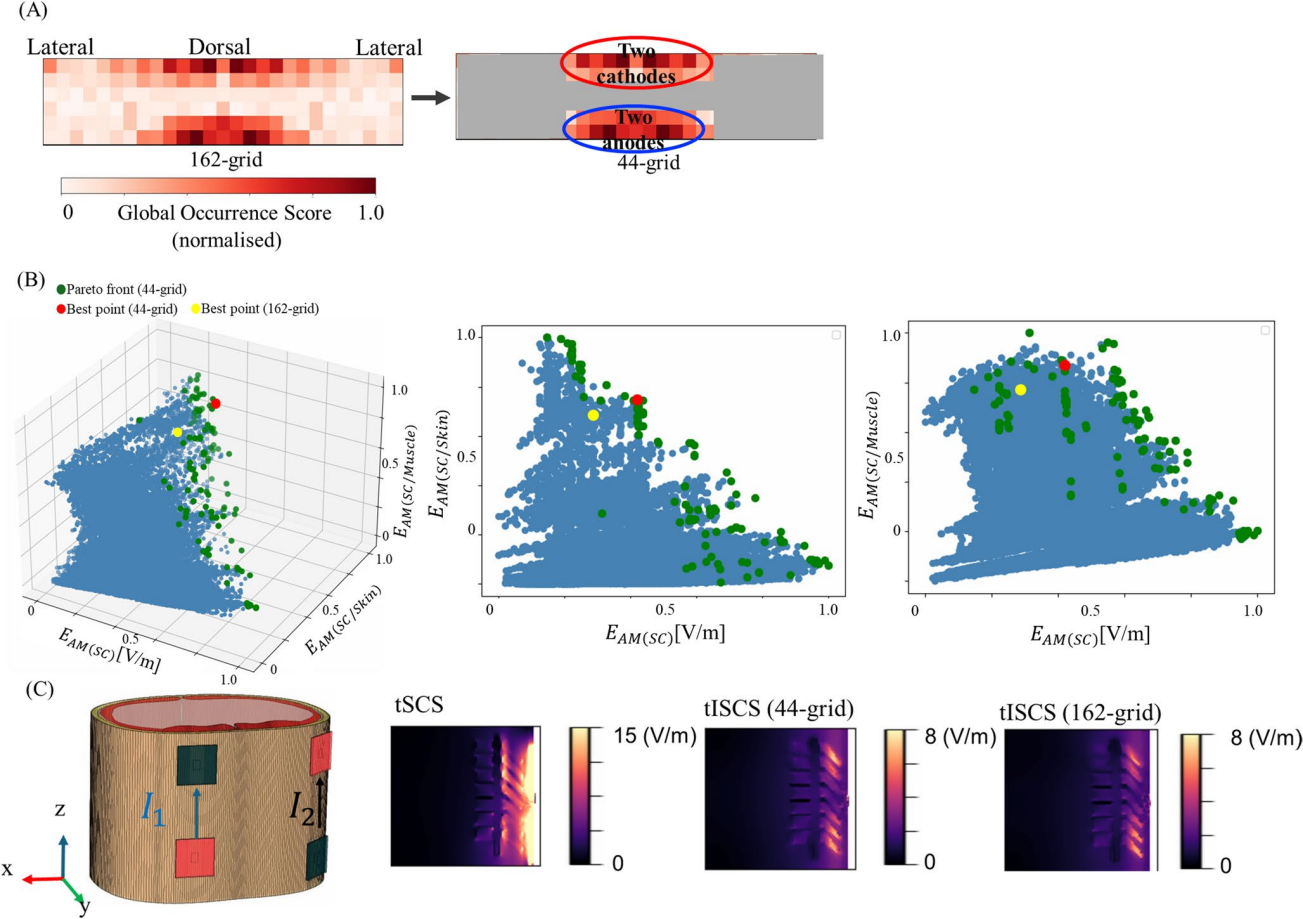
tISCS optimal montage with reduced grid space (44-grid). **A** Most dominant electrode positions, **B** local best montage from a total of 50,000 random selected montages based on Pareto fronts (3D). The same results of the 3D pareto front is also shown from two 2D perspectives

We focus on identifying the most influential electrode positions to narrow the search space. A Global Relevant Electrode Map was created by integrating data from the six trials, as shown in Fig. 5A, following the procedure outlined in Fig. 3B. The color intensity on the map reflects the frequency of electrode utilization, with more prominent colors indicating positions associated with favorable outcomes. The results show that electrodes in the central dorsal region were predominantly used. As a result, the number of potential electrode locations (search space) was reduced from 162 to 44 considering a threshold over the map of 0.5 covering electrodes on the back.

Figure 5B illustrates the optimization based on the 44-grid. The yellow point represents representative trial in Sect. 3.1, which is based on the 162-grid, while the red point indicates the best montage based on the 44-grid, which is closer to the ideal point. Figure 5C displays the peak interferential electric field distribution ${E}_{AM}$ within the thorax model. As indicated in Table 3, the locally best montage using the 44-grid improved spinal cord intensity by 1.2 times compared to the best 162-grid montage, while enhancing the spinal cord-to-muscle ratio by 1.1 and reducing spinal cord-to-skin ratios by 1.1. These findings suggest that refining electrode positions using the Global Relevant Electrode Map enhances the optimization process for tISCS.

**Table 3:** The comparison of E-field magnitude between tSCS and tISCS

Metrics	tSCS	2-tSCS	tISCS (44-grid)	tISCS (162-grid)
Electric field in spinal cord (V/m)	4.24	2.67	2.51	2.13
Spinal cord / skin	0.03	0.04	0.39	0.35
Spinal cord / muscle	0.19	0.24	0.70	0.63

Furthermore, tISCS (44-grid) improves the metrics compared to tSCS and 2-tSCSs. Compared to 2-tSCS, the spinal cord intensity under tISCS decreases by a factor of 1.1 times while the spinal cord-to-skin and spinal cord-to-muscle ratios remain higher by 9.8 and 2.9 times, respectively. Compared to tSCS, the spinal cord intensity under tISCS decreases by a factor of 1.7 times while the spinal cord-to-skin and spinal cord-to-muscle ratios remain higher by 13.0 and 3.7 times, respectively.

## Discussion

Transcutaneous spinal cord stimulation (tSCS) has been investigated for its applications in pain management, motor function rehabilitation, and the treatment of chronic and neuropathic pain. However, tSCS faces challenges in accurately targeting specific nerve regions due to its low focality and significant current attenuation when penetrating deeper areas of the spinal cord. Consequently, higher electrical currents are often needed, which can activate nociceptors and induce pain, while also potentially stimulating nearby organs, including muscles. This work presents the first computational demonstration of a novel non-invasive neuromodulation technique, transcutaneous interferential spinal cord stimulation (tISCS), which utilizes temporal interference to achieve deeper and more selective activation of spinal cord circuits. Using a finite element model combined with a leadfield-based Pareto optimization strategy, we show that tISCS significantly enhances focality and reduces off-target stimulation compared to traditional transcutaneous spinal cord stimulation (tSCS).

The results of our study demonstrate that tISCS reduces off-target skin intensities by at least a factor of 20 compared to tSCS. This reduction is crucial as it minimizes discomfort. Moreover, the spinal cord-to-skin electric field ratio in tISCS is at least 10 times higher than in either 2-tSCS or tSCS. This ratio enables a sevenfold increase in spinal cord stimulation intensity without increasing off-target stimulation, even when accounting for the approximately twofold reduction in spinal cord field strength inherent to tISCS. Importantly, with a total injection current of 2.5 mA, tISCS still achieves a minimum electric field threshold for neuromodulation (0.35 V/m) [41]. The optimization was based on a Pareto front representing the trade-off between maximizing the electric field in the spinal cord and increasing the spinal cord-to-off-target tissue ratio. Notably, skin tissue served as a more effective off-target constraint, aligning with the lower activation thresholds of non-nociceptive fibers compared to muscle fibers.

Our approach also addresses the constraint on computation time. By using the lead-field matrix, each montage is solved in 80 seconds, which is faster than previously reported times (e.g., 2.5 h per montage, 26 million DOF, COMSOL Multiphysics [18]). Moreover, using a reduced electrode position space (44-grid) enabled a more efficient exploration of potential electrode placements without compromising computational time. It was demonstrated that optimization at more relevant positions improved the trade-off between intensity in the spinal cord and focality.

The current study has several limitations. Major potential inaccuracies in electric field FEM simulations stem from anatomical approximations and assumptions about tissue conductivity. Firstly, our model acts as a proxy for an anatomical model representing the lower thorax, given that simplified models capture the complexities of more detailed models [42]. Future research should consider subject-specific modelling (e.g., based on MRI or other high-resolution images) to investigate inter-individual differences in the effects of tISCS in clinical research applications. In such a case, the leadfield approach should be applied on a subject-specific basis, and integrating it with other optimization methods could further accelerate the process [43]. Secondly, while the selection of conductivity values may affect absolute peak magnitudes, it does not significantly influence the relative spatial distribution of the electric field and is therefore unlikely to hinder the optimization outcome [44]. Finally, we relied on the quasi-static assumptions, which include neglecting dispersion phenomena, and treating the medium as purely resistive without considering capacitive effects. Relaxing the quasi-static assumptions can result in differences of up to 20% in model fiber responses to kilohertz stimuli [49]. Therefore, more precise estimation of the temporal dynamics of the electric field may be determinant for optimization strategies focused on combined assessment of electrode placement and stimulation waveform [45] [46]. As future work, magnetic stimulation is a modality that induces a transverse electric field in cutaneous nociceptive fibers, which may improve tolerability compared with tSCS. Future studies directly comparing tISCS and magnetic stimulation [47] will be valuable for clarifying their relative efficacy and tolerability.

## Conclusion

This study presents a computational analysis aimed at optimizing transcutaneous interference spinal cord stimulation (tISCS). Results show that tISCS significantly reduces off-target stimulation in superficial tissues and enhances spinal cord-to-skin connectivity compared to conventional tSCS. A leadfield matrix alleviated computational challenges and shortened simulation time, enabling the evaluation of an extensive electrode position search that could be improved by adopting a refined montage space based on more influential positions. This study advances tISCS as a promising non-invasive alternative to traditional spinal cord stimulation, allowing for stronger stimulation without compromising patient comfort.

## Supplementary Information

Below is the link to the electronic supplementary material.

Supplementary Material 1

## Data Availability

The data supporting the findings of this study are available from the corresponding author upon reasonable request.
