# Supplementary material for: Transcutaneous interference spinal cord stimulation: leadfield-based pareto optimization of electrode montages for improved focality
Source: Biomed Eng Lett. 2025 Nov 17;16(2):483–92. doi: 10.1007/s13534-025-00531-2 (PMC13013866; doi:10.1007/s13534-025-00531-2)
Supplement: Supplementary file 1 — Supplementary Material 1 [file 13534_2025_531_MOESM1_ESM.docx]

**Supplementary Material: Stability of Optimization Method**

In the first step, to approximate the optimal montage, we employed a brute-force search randomly sampled subsets of the full montage space (“quasi-optimization”), enabling unbiased global exploration. To assess the stability of this procedure, we varied the sampled subspace size from 5,000 to 50,000 candidates and, for each size, performed six independent random trials (Table 1).

Results show two consistent trends. First, the mean spinal-cord modulation depth increases slowly with subspace size, reflecting the higher probability of sampling better montages as coverage grows. Second, focality ratios converge with increasing subspace size. Quantitatively, the percent change between consecutive sizes was <6% from 10,000 to 20,000 and <10% from 20,000 to 50,000 (Fig. 1). In addition, the standard deviation across the six repeated random trials also decreases with size and plateaus at 10,000 (Table 1). Taken together, these findings indicate that subspaces larger than 10,000 provide a reliable and computationally efficient global search, balancing accuracy with runtime.

In the second step, we restricted the search to the most influential electrodes identified during global exploration (the “relevant electrode map” in subsection 2.6), reducing the grid from 162 to 44 positions, and then performed an exhaustive evaluation over all montage combinations in this reduced space.

**Table 1:** Effect of subspace size in the quasi-optimization method (*n* = 6)

| Subspace Size | Average Electric Field [V/m]  (Relative SD [%]) | Average Focality Ratio  (Relative SD [%]) | |
| --- | --- | --- | --- |
|  | Spinal Cord | Spinal Cord / Skin | Spinal Cord / Muscle |
| 1,000 | 1.28 (23%) | 0.25 (35%) | 0.56 (46%) |
| 5,000 | 1.82 (23%) | 0.30 (18%) | 0.43 (27%) |
| 10,000 | 1.65 (18%) | 0.36 (17%) | 0.56 (6%) |
| 20,000 | 1.76 (15%) | 0.34 (20%) | 0.53 (9%) |
| 50,000 | 1.84 (18%) | 0.37 (17%) | 0.55 (6%) |


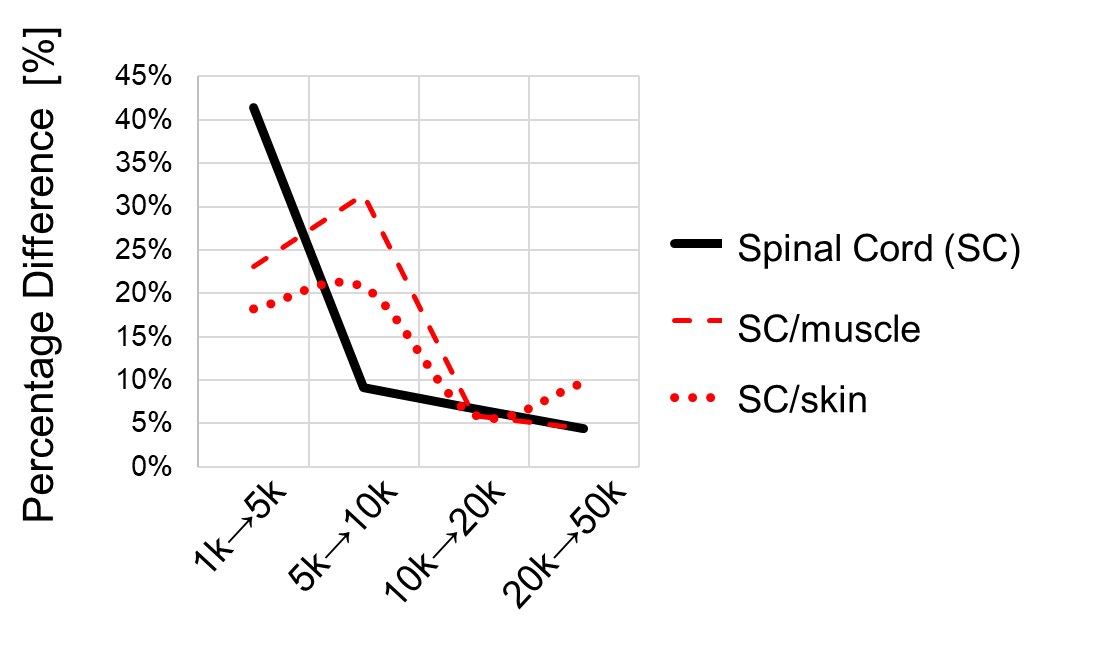


**Figure 1** Percentage difference between subspace sizes
